# Supplementary material for: Evolution of Asian Corn Borer Resistance to Bt Toxins Used Singly or in Pairs
Source: Toxins (Basel). 2019 Aug 6;11(8):461. doi: 10.3390/toxins11080461 (PMC6723947; doi:10.3390/toxins11080461)
Supplement: Supplementary file 1 [file toxins-11-00461-s001.pdf]

Supplementary Materials: Evolution of Asian Corn Borer Resistance to Bt Toxins used Singly or in Pairs

Yueqin Wang, Yudong Quan, Jing Yang, Changlong Shu, Zhenying Wang, Jie Zhang, Angharad M. R. Gatehouse, Bruce E. Tabashnik and Kanglai He

Table S1. Resistance ratios (RRs) and their 95% confidence intervals (CIs) for five strains of *Ostrinia furnacalis* selected with Bt toxins singly or in pairs.

Three strains selected with a single toxin					Two strains selected with a pair of toxins									
Selected			95% CI		Selected			95% CI		Selected			95% CI	
with	Gen.	RR	Lower	Upper	with	Gen.	RR	Lower	Upper	with	Gen.	RR	Lower	Upper
		**Cry1Ab**					**Cry1Ab**					**Cry1Ab**		
**Cry1Ab**	1	1.4	0.8	2.5	**Cry1Ab**	1	2.0	1.4	2.9	**Cry1Ab**	1	1.6	1.1	2.3
	2	1.1	0.7	1.7	**+ Cry1F**	2	3.7	3.0	4.5	**+Cry1Ie**	2	1.6	1.2	2.2
	3	0.9	0.6	1.5		3	3.3	2.6	4.1		3	1.9	1.4	2.5
	4	3.9	2.4	6.4		4	3.8	2.9	5.0		4	3.0	2.4	3.8
	5	4.7	3.5	6.3		5	2.7	2.0	3.7		5	3.0	2.2	4.1
	6	5.8	4.5	7.5		6	7.8	5.8	10.6		6	7.6	5.7	10.1
	7	13.2	10.1	17.2		7	17.9	13.6	23.6		7	15.6	11.0	22.0
	8	18.5	14.9	23.0		8	17.6	13.6	22.8		8	14.7	10.7	20.2
	9	23.4	18.2	30.0		9	11.2	8.8	14.3		9	24.3	18.2	32.6
	10	23.6	17.2	31.3		10	12.5	9.0	17.4		10	29.8	21.1	42.2
	11	32.0	24.3	42.2		11	32.1	24.6	42.0		11	44.6	33.2	59.8
	12	38.8	30.8	48.9		12	35.2	27.1	45.7		12	53.8	42.2	68.7
	13	33.1	22.9	47.8		13	36.8	28.9	46.9		13	64.6	51.2	81.5
	14	28.2	22.6	35.2		14	32.5	23.4	45.1		14	60.4	46.7	78.5
		**Cry1F**					**Cry1F**							
**Cry1F**	1	1	0.8	1.3	**Cry1Ab**	1	1.5	1.1	2					
	2	1.5	1.1	2.0	**+ Cry1F**	2	2.0	1.5	2.8					
	3	1.4	1.1	1.8		3	1.8	1.4	2.4					
	4	2.6	1.8	3.9		4	5.8	4.5	7.4					
	5	59.0	44.7	77.9		5	45.4	28.8	71.6					
	6	557	419	740		6	240	167	344					
	7	>600				7	>600							
	9	>1300				9	-							
	10	>1250				10	-							
	11	>1250				11	>1250							
	12	>1250				12	-							

	13	>1250			13	-					
	14	>1250			14	>1250					
		**Cry1Ie**							**Cry1Ie**		
**Cry1Ie**	1	0.6	0.5	0.7			**Cry1Ab**	1	2.6	2	3.4
	2	0.8	0.7	1.0			**+Cry1Ie**	2	2.7	1.9	3.9
	3	0.9	0.6	1.5				3	2.4	1.9	3
	4	3	2.3	4.0				4	10.2	7.3	14.4
	5	54.9	42.2	71.5				5	85.4	62.6	116.4
	6	111	77.3	160				6	>400		
	7	>400						7	>400		
	9	>800						9			
	10							10	>830		
	12	>1000						12			
	14	>840						14	>1100		

Table S2. LC_{50}s and their 95% fiducial limits (in μg toxin per g diet) for five strains of *Ostrinia furnacalis* selected with Bt toxins singly or in pairs.

**Three strains selected with a single toxin**				**Two strains selected with a pair of toxins**							
**Selected with**	**Gen.**	**n**	**LC50 (95% FL)***	**Selected with**	**Gen.**	**n**	**LC50 (95% FL)**	**Selected with**	**Gen.**	**n**	**LC50 (95% FL)**
			**Cry1Ab**								**Cry1Ab**
**Cry1Ab**	0	672	0.21 (0.16–0.27)	**Cry1Ab**	0	480	0.18 (0.14–0.22)	**Cry1Ab**	0	480	0.18 (0.14–0.22)
	1	720	0.27 (0.05–0.41)	**+ Cry1F**	1	480	0.39 (0.19–0.58)	+ Cry1Ie	1	480	0.30 (0.19–0.40)
	2	720	0.22 (0.13–0.35)		2	480	0.71 (0.59–0.81)		2	480	0.31 (0.21–0.42)
	3	720	0.17 (0.08–0.29)		3	384	0.64 (0.52–0.75)		3	384	0.37 (0.24–0.50)
	4	480	0.75 (0.48–1.42)		4	432	0.74 (0.58–0.92)		4	576	0.59 (0.49–0.70)
	5	480	0.90 (0.69–1.14)		5	576	0.52 (0.39–0.67)		5	528	0.58 (0.43–0.74)
	6	528	1.12 (0.89–1.37)		6	720	1.52 (1.15–1.94)		6	816	1.48 (1.15–1.87)
	7	480	2.57 (2.02–3.14)		7	624	3.48 (2.69–4.30)		7	624	3.02 (2.11–3.98)
	8	528	3.59 (3.07–4.21)		8	624	3.42 (2.74–4.18)		8	576	2.86 (2.12–3.73)
	9	576	4.55 (3.69–5.51)		9	480	2.18 (1.79–2.62)		9	528	4.72 (3.58–5.97)
	10	576	4.58 (3.52–5.72)		10	528	2.45 (1.77–3.12)		10	528	5.79 (4.01–7.66)
	11	576	6.21 (4.80–7.70)		11	576	6.23 (4.94–7.72)		11	624	8.65 (6.56–10.9)
	12	576	7.53 (6.32–9.01)		12	624	6.84 (5.39–8.31)		12	624	10.5 (8.44–13.3)
	13	672	6.10 (4.91–7.36)		13	624	7.14 (5.84–8.59)		13	672	12.5 (10.5–15.0)
	14	576	5.49 (4.42–6.80)		14	672	6.32 (4.61–8.26)		14	624	11.7 (9.38–14.3)
			**Cry1F**				**Cry1F**				
**Cry1F**	0	720	0.54 (0.40–0.69)	**Cry1Ab**	0	576	0.54 (0.27–0.84)				
	1	720	0.80 (0.64–0.97)	**+ Cry1F**	1	432	1.15 (0.71–1.68)				
	2	720	1.16 (0.93–1.44)		2	480	1.61 (1.21–2.10)				
	3	720	1.07 (0.89–1.28)		3	384	1.45 (1.14–1.80)				
	4	480	2.09 (0.86–3.02)		4	576	4.57 (3.79–5.42)				
	5	528	48.2 (38.6–59.1)		5	672	36.1 (23.7–54.9)				
	6	672	442 (366–604)		6	672	190 (143–269)				
	7	96	>476 (7.5%)		7	96	> 476 (6.5%)				
	9	96	>1030 (48.4%)		9						
	10	96	>1000 (23.5%)		10						
	11	96	>1000 (35.7%)		11	96	> 1000 (47.3%)				
	12	96	>1000 (47.3%)		12						
	13	96	>1000 (27.9%)		13						
	14	96	>1000 (41.7%)		14	96	> 1000 (46.8%)				
			**Cry1Ie**								**Cry1Ie**

**Cry1Ie**	0	576	1.25 (1.07–1.42)	**Cry1Ab + Cry1Ie**	0	480	2.45 (1.73–3.35)
	1	576	1.55 (1.32–1.75)		1	480	6.27 (4.94–7.80)
	2	576	1.84 (1.61–2.05)		2	480	6.56 (4.52–8.52)
	3	480	2.19 (0.77–4.14)		3	384	5.78 (4.80–6.85)
	4	480	7.39 (5.72–9.32)		4	576	24.9 (18.9–35.1)
	5	480	133 (107–166)		5	672	207 (159–274)
	6	576	270 (198–397)		6	96	> 952 (12.9%)
	7	96	>952 (8.6%)		7	96	> 952 (14.1%)
	9	48	>1935 (10.4%)		9		
	10				10	48	>2020 (8.7%)
	12	48	>2480 (0.0%)		12		
	13				13	48	>2700 (4.2%)
	14	96	>2047 (0.0%)		14	48	>2680 (0.0%)

*When the highest concentration tested killed <50% of larvae, the LC_{50} is listed as > the highest concentration tested, the percentage mortality at that concentration in parentheses (adjusted for control mortality), and n is the number of larvae tested at that concentration, for example, in generation 7 for the strain selected with Cry1F, 7.5% of larvae were killed by 476 μg Cry1F per g diet, the highest concentration tested.

Table S3. LC_{50}s and their 95% fiducial limits (in μg toxin per g diet) for the unselected susceptible S strain of *Ostrinia furnacalis*.

Replicate [a]	n	LC50 (95% FL)
		**Cry1Ab**
**A**	480	0.18 (0.14–0.22)
**B**	480	0.18 (0.14–0.22)
**C**	576	0.23 (0.17–0.30)
**D**	576	0.17 (0.14–0.21)
**E**	576	0.21 (0.17–0.25)
**F**	672	0.22 (0.14–0.30)
**Pooled**	**3360**	**0.19 (0.16–0.23)**
		**Cry1F**
**A**	576	0.54 (0.27–0.84)
**B**	480	1.00 (0.80–1.24)
**C**	576	1.17 (0.90–1.48)

**D**	576	0.60 (0.46–0.76)
**E**	624	0.75 (0.58–0.95)
**F**	672	0.74 (0.58–0.92)
**Pooled**	**3504**	**0.79 (0.63–0.96)**
		**Cry1Ie**
**A**	480	2.45 (1.73–3.35)
**B**	384	2.62 (2.22–3.06)
**C**	528	2.68 (2.15–3.26)
**D**	528	2.01 (1.60–2.47)
**E**	624	1.96 (1.52–2.45)
**F**	528	2.21 (1.74–2.77)
**G**	576	2.34 (1.87–2.88)
**Pooled**	**3648**	**2.43 (2.07–2.79)**

[a] Replicates were done throughout the 14 generations of the selection experiment, roughly every other generation.
